# Immune Modulations by Glucocorticoids: From Molecular Biology to Clinical Research

**DOI:** 10.3390/cells11244032

**Published:** 2022-12-13

**Authors:** Marcel J. M. Schaaf, Onno C. Meijer

**Affiliations:** 1 Institute of Biology, Leiden University, Einsteinweg 55, 2333 CC Leiden, The Netherlands; 2 Department of Endocrinology, Leiden University Medical Center, Albinusdreef 2, 2333 ZA Leiden, The Netherlands

Due to their potent anti-inflammatory and immune-suppressive actions, glucocorticoids have been used in the treatment of inflammatory and autoimmune disease for more than 70 years. They are highly effective drugs, but their use is limited by the severity of their side effects, which include osteoporosis, muscle wasting, hyperglycemia and hypertension, and by the occurrence of resistance to glucocorticoid therapy. In this Special Issue, an overview is presented of our current understanding of the mechanisms underlying the therapeutic and side effects of glucocorticoid treatment, as well as the decreased sensitivity observed in resistant patients. In addition, research is highlighted that either aims to develop novel glucocorticoid therapies with reduced side effects, or prevent or treat glucocorticoid resistance.

The anti-inflammatory effects of glucocorticoids are mediated by an intracellular receptor, the glucocorticoid receptor (GR). Over the last two decades, research in which the use of cell type-specific GR knockout mice was combined with infectious, autoimmune and inflammatory disease models has provided a wealth of data on which cells form the primary target of the therapeutic effects of glucocorticoids. Two review articles, by Rocamora-Reverte et al. [1] and Reichardt et al. [2], discuss the results of these studies, which have revealed that the immune-modulating effects of glucocorticoids are remarkably cell type- and disease model-dependent. This appears to be true not only for the therapeutic effects of administered glucocorticoid drugs on the disease, but also for the effects of the endogenous glucocorticoid corticosterone on immune function. Taking together the results of these studies, a picture emerges in which glucocorticoids may target a variety of cell types within the immune system, including innate lymphoid cells, T- and B-cells, myeloid cells (granulocytes, macrophages and dendritic cells), and other immune cells, as well as various non-immune cell types, including epithelial and stromal cells.

Interestingly, the primary target cell that mediates the anti-inflammatory action of glucocorticoid action differs largely between disease models. In models for inflammatory bowel disease, graft-versus-host disease, neuroinflammation, and in an antigen-induced arthritis model, T cells were shown to be indispensable for the glucocorticoid effects. In contrast, T cells were demonstrated not to be involved in glucocorticoid action in other (collagen- and serum-transfer induced) arthritis models and in models for contact dermatitis. In this latter model, myeloid cells appeared to play a crucial role, and these cells were also indispensable in a model for acute lung injury. In a model for allergic asthma, none of the cell types from the immune system appeared to be involved in glucocorticoid action, and the primary target was shown to be a stromal cell type. A similar cell type, which strongly interacts with macrophages, was also shown in mediating glucocorticoid effects in the collagen- and serum-transfer induced arthritis models.

In the different cell types that are targeted by glucocorticoids, these steroids bind the GR, which subsequently becomes activated and acts as a transcription factor, regulating the expression of a plethora of genes. The GR alters the transcription rate of these genes through several mechanisms of action, some of which require dimerization of the receptor. As a homodimer complex, it can directly bind to glucocorticoid response elements (GREs) in the DNA to transactivate genes. Alternatively, it can bind to negative GREs and repress gene transcription, or to composite elements, which contain a (half) GRE and a response element of another transcription factor, and GR binding may either activate or repress transcription. As a monomer, the GR can also indirectly bind to DNA by physically interacting with other transcription factors, thereby modulating their activity. Classically, monomeric GRs tethering to pro-inflammatory transcription factors, such as NF-κB and AP-1, were considered to mediate the anti-inflammatory action of glucocorticoids, whereas dimeric GRs transactivating gene expression through GRE binding were considered responsible for side effects of glucocorticoid treatment, such as hyperglycemia. However, in recent years it has become clear that the picture is more complex.

In a review article, Timmermans et al. [3] describe our current understanding of the (patho-)physiological role of GR dimer formation, and illustrate that the anti-inflammatory effects of glucocorticoids and their side effects cannot be simply distinguished based on the monomeric and dimeric GR conformations, respectively. Most of these insights are based on in vitro and in vivo studies in which the action of a mutant GR is investigated that is deficient in dimerization, as a result of a point mutation in the dimerization interface in the DNA binding domain. Although there is controversy about whether this GR^dim^ mutant is completely dimerization-deficient and it has been shown to actually bind DNA and even induce increased GRE-dependent transactivation of a small number of genes, this mutation generally shifts the balance of GR-mediated gene regulation to dimerization-independent mechanisms such as tethering. In several mouse models for inflammatory diseases, the therapeutic glucocorticoid effect is intact in GR^dim/dim^ mice, which is in line with a major role played by monomeric GR. However, these mutant mice show a strongly reduced response to glucocorticoids in other models, especially those mimicking acute inflammatory conditions such as systemic inflammatory response syndrome and sepsis. This observation suggests that GR dimers represent the main mechanism mediating the therapeutic glucocorticoid action in these situations and are thought to elicit these effects by transactivating genes encoding anti-inflammatory proteins, as well as by suppressing the transcription of genes encoding pro-inflammatory genes—for example, by binding to specific nGREs. To make the picture even more complex, certain side effects, such as osteoporosis, were shown to be independent of receptor dimerization.

An interesting and original approach to more selectively target anti-inflammatory effects is provided by Greulich et al. [4], who focused on the regulation of enhancer RNAs (eRNAs) in macrophages. In contrast to mRNAs, GR binding in most cases was associated with eRNA synthesis, mostly in intragenic areas of the chromatin. The eRNA induction correlated with mRNA changes in nearby genes. Central to the argumentation of the authors is the fact that eRNAs showed an even more pronounced cell-type specific pattern compared to mRNAs. Although it is too early to draw firm conclusions, the authors speculate that these eRNAs (perhaps in the context of ‘concentrating’ nuclear condensates) may contribute to cell-specific responses and eventually be used to achieve higher cell-type specificity in anti-inflammatory effects.

The induction of osteoporosis by glucocorticoid treatment was investigated by Palmowski et al. [5]. In their research article, they present the results of a study on 198 patients suffering from either polymyalgia rheumatica or a type of vasculitis, with most of them being treated with glucocorticoids. Surprisingly, the authors found no association between glucocorticoid treatment and the bone mineral density of these patients. Interestingly, they did find an association between a low bone density and treatment with proton pump inhibitors, which are often used to mitigate the risk of glucocorticoid-induced gastric ulcer formation. According to the authors, this latter finding should be given more attention.

Although it is now clear that newly developed glucocorticoids that induce a GR conformation that is unable to dimerize will not entirely dissociate the anti-inflammatory action from the side effects, researchers are still developing so-called Selective GR Agonists and Modulators (SEGRAMs) based on the idea that partial dissociation will be enough to sufficiently shift the therapeutic index. Several steroidal and non-steroidal SEGRAMS have been developed, such as mapracorat and vamorolone, which are currently being tested in clinical trials. An overview of SEGRAM development is presented by Reichardt et al. in their review article [2], in which they also discuss a second approach that is being used to reduce the side effects of glucocorticoids—the use of nanoformulations that improve drug targeting to specific tissues or cell types. The encapsulation of glucocorticoids in several types of liposomes (vesicles formed by a lipid bilayer) has been shown to improve their therapeutic efficacy in many preclinical models, and some of these formulations have even reached the clinical trial stage. A research article by Xie et al. [6] describes the use of zebrafish embryos for the screening of novel liposome formulations. Using this model, they show that PEGylated liposomes remain in circulation for long periods of time, whereas a novel type of liposomes selectively targets macrophages. Both types of liposomes increased the therapeutic ratio of the encapsulated drug in their model. Besides liposomes, novel inorganic–organic hybrid nanoparticles and several other nanoparticle formulations, such as poly-δ-decalactone/methoxy-polyethylene glycol- and cyclodextrin-based nanoparticles and modified exosomes, have been developed to carry glucocorticoids. The large variety of these approaches and the positive results of many preclinical investigations involving these nanoparticles provides confidence that many of these formulations will be tested in clinical studies in the near future.

Another approach to reduce the side effects of glucocorticoids is described in a research article by Zappia et al. [7]. They show that antihistamines potentiate both the glucocorticoid-induced suppression of pro-inflammatory gene transcription and the activation of anti-inflammatory gene transcription in two cell lines. This potentiation is mediated by the histamine H1 receptor and appears to be remarkably ligand-, cell type-, and gene-specific. It was expected that the enhancement of glucocorticoid-induced transcriptional changes associated with side effects would also be potentiated. However, antihistamine treatment counteracted the glucocorticoid effects on bone metabolism marker genes, which suggests that glucocorticoid-induced osteoporosis may not be enhanced, but even prevented by the cotreatment. Therefore, the authors suggest that cotreatment with antihistamines may allow lowering the dose of glucocorticoid treatment, thereby reducing its side effects.

The risks and benefits of glucocorticoid use are perhaps most poignantly explicit in cancer therapy, as reviewed by Kalfeist et al. [8]. Glucocorticoids are used very often in cancer patients. In immune cancers they are a key aspect of treatment due to their immune-suppressive effects, and here, the development of glucocorticoid resistance is also a serious potential problem. In many other cancer types, glucocorticoids are used to ameliorate the side effects of chemotherapy or the consequences of the disease. Yet, in particular cancers, glucocorticoids seem to support tumor survival or metastasis formation. With the advance of immune therapy, glucocorticoid-induced reduced immune responses may actually work against successful therapy, even if glucocorticoids may also ameliorate its side effects. The use of combinations of glucocorticoids and immune therapy and the possible timing thereof are still under debate and form an interesting area of research that will certainly develop in the coming years.

The occurrence of GR-induced side effects is particularly problematic when therapeutic effects attenuate over time. Such GR resistance is a clinical issue and may depend on several mechanisms. First, there are intrinsic differences in GR responsiveness based on genetic variation. Complete GR resistance is very rare, but more common variants may contribute to a risk of disease and to responsiveness to glucocorticoid treatment. Pac et al. [9] show that single nucleotide polymorphisms (SNPs) in the GR gene are associated to IgA nephropathy (IgAN) and membranous nephropathy (MN), which are both immune-mediated glomerular kidney diseases, in which glucocorticoid resistance may prevent successful disease management. They found that the gain-of-function ‘Bcl1’ variant was associated with lower disease susceptibility and particular histopathological aspects of the disease. They also found associations of the Rs17209237 SNP with disease progression, both in steroid-sensitive and -insensitive patients. These outcomes underline the importance of genetic background when discussing GR’s role in the pathogenesis of (auto)immune diseases and the glucocorticoid treatment of these diseases.

GR levels may be limiting for the response to glucocorticoids, and low GR levels may tamper with the effects of endogenous hormones, as well as play a role in resistance to exogenous glucocorticoid treatment. Spies et al. [10] provide an overview of the current knowledge on the regulation of GR levels, with a focus on homologous downregulation. GR downregulation occurs at the transcriptional level and through miRNAs, and this directly links it to the differential effects of SEGRAs. GR degradation is also intrinsically linked to the ubiquitin-proteosomal system. Importantly, GR protein degradation is not only ligand-dependent, but is also regulated by phosphorylation. Moreover, the activity of the many components of the ubiquitination dependent proteosomal degradation pathways may differ per cell type or condition. While it is clear from patients with Cushing’s disease that GR downregulation may dampen but not fully prevent the consequences of hypercortisolism, the homologous downregulation via the more potent synthetic glucocorticoids may contribute to the development of glucocorticoid resistance.

However, there is more to GR resistance than the levels of functional GR protein, and cross-talk with other signaling pathways is likely to be as important. A good case has been made for hypoxia-induced factors, or HIFs, a class of transcription factors that become active under conditions of reduced environmental oxygen availability, which may occur in inflamed tissues. In their review, Marchi et al. [11] discuss extensive evidence for a complex cross-talk between HIFs and GR. In zebrafish and mouse studies, GR seems to potentiate HIF responses, while HIF activation suppresses both cortisol synthesis and redirects genomic GR responsiveness, effectively leading to curbed GR responses for many genes.

In order to better predict when patients become resistant to glucocorticoid therapy and to be able to switch to a more effective therapy in time, validated biomarkers are necessary. In an elaborate study encompassing experiments on human patient samples, a mouse model, organoids and cell cultures, Landskron et al. [12] investigated the relationship between glucocorticoid responsiveness and the induction of local cortisol production in the intestines of ulcerative colitis patients. They found increased cytoplasmic levels of the transcription factor LRH-1—which is known to be involved in intestinal steroidogenesis—in the intestinal cells of steroid-dependent and steroid-refractory patients, providing a possible explanation for the decreased local cortisol production in these patients. In addition, they found elevated levels of GRβ, a truncated GR variant that interferes with signaling of the canonical GR, in the intestines of these patient groups. The authors therefore suggest performing further studies to confirm the LRH-1/GRβ profile as a biomarker for glucocorticoid responsiveness.
